# Nematodes in the Pine Forests of Northern and Central Greece

**DOI:** 10.3390/insects13020194

**Published:** 2022-02-13

**Authors:** Maria Karmezi, Alkmini Bataka, Dimitrios Papachristos, Dimitrios N. Avtzis

**Affiliations:** 1 Forest Research Institute, Hellenic Agricultural Organization Demeter, Vassilika, 57006 Thessaloniki, Greece; alkminimp@fri.gr (A.B.); dimitrios.avtzis@fri.gr (D.N.A.); 2 Zoology Department, School of Biology, Faculty of Sciences, Aristotle University of Thessaloniki, 54124 Thessaloniki, Greece; 3 Scientific Directorate of Entomology and Agricultural Zoology, Benaki Phytopathological Institute, Kifissia, 14561 Athens, Greece; d.papachristos@bpi.gr

**Keywords:** *Bursaphelenchus* spp., national survey program, nematodes, conifers, Greece

## Abstract

**Simple Summary:**

Pine wood nematode *Bursaphelenchus xylophilus* is the agent of pine wilt disease and one of the most important forest tree pathogens worldwide, transmitted through beetles of the *Monochamus* genus. As an invasive species, it has spread beyond its natural range by human activity mainly wood trade. The devastating impact it has on pine forests has led to severe environmental and economic damages in its introduced countries. The wide distribution of *Monochamus* spp. beetles in many parts of the world along with favourable climatic conditions, which are both important factors for the establishment of pine wilt disease, have raised awareness over its continuous expansion. Therefore, in an attempt to control and even inhibit its further spread and consequently its severely adverse impacts, appropriate measures have already been taken and implemented from countries across the globe.

**Abstract:**

In the context of plants or plant products protection by harmful organisms, measures have been taken by EU countries in order to prevent their introduction and establishment into the EU, and also limit their expansion in case they do enter. Such a case is *Bursaphelenchus xylophilus* (Parasitaphelenchidae, Nematoda), already recorded in Portugal and Spain. So, Member States should take all the appropriate steps in order to monitor and confine if necessary susceptible plants and/or plant products. Such measures include annual surveys even in countries where pine wilt disease does not occur yet. Therefore, national survey programs are widely established, sampling and examining samples from pine trees showing suspicious symptoms that could potentially be attributed to *B. xylophilus*. In this direction, such a network has also been established in Greece collecting and examining wood samples nationwide. In total, 123 wood samples were collected from conifer trees of Northern and Central Greece. Though *B. xylophilus* was absent from all samples examined, four other *Bursaphelenchus* species were identified. In addition, other nematode taxa were also recorded, including several phytophagous, microbivorous as well as predatory nematode species. This highlights the fact that besides preventing the introduction of *B. xylophilus* in Greece, national survey programs can significantly contribute to and enhance our knowledge of the indigenous nematode species.

## 1. Introduction

The pine wood nematode (PWN) *Bursaphelenchus xylophilus* Steiner & Buhrer 1934 is one of the most important pathogens worldwide [1] that causes pine wilt disease (PWD), and it is currently included in the EPPO A2 list of pests that are recommended for regulation as quarantine pests [2]. *B. xylophilus* natural pathway of transport between hosts is by the adult stages of the longhorn beetle of the genus *Monochamus* (Coleoptera, Cerambycidae). *B. xylophilus* is transmitted either during maturation feeding on healthy trees (primary transmission) or during oviposition on weakened and susceptible trees (secondary transmission) [3]. PWN dispersal juveniles (“dauer” larvae) are carried mainly within the respiratory system (tracheae) of *Monochamus* spp. beetles. During maturation feeding (phytophagous phase), PWN is transmitted on healthy pine trees where it spreads in the vascular system of the tree and resin canals. There it feeds on epithelial cells and living parenchyma causing a rapid reduction in the complete cessation of the resin flow. Cell destruction leads to embolism of the tracheids, blocking water conductance (cavitation) tree’s death, or dead trees attract female insects for oviposition and nematodes enter the tree by oviposition slits in the bark (mycophagous phase). *Monochamus* spp. larvae burrow into the wood where nematodes surround the pupal chambers and enter into the insect’s body through openings such as the spiracles. The transmission cycle continues through the maturation feeding of the young immature adult *Monochamus* insects. [3,4,5]. However, the high risk of introduction of the pine wood nematode into other countries, revealed in the relevant Pest Risk Analysis [6], is significantly magnified by human-mediated activities, following the routes and pathways employed by international wood trade either as a commodity or as wood packaging material (WPM) [7].

The pine wood nematode is indigenous in North America (the US and Canada) [8] where native pine species are relatively tolerant to its infestation. That was not the case in Japan, however, the first country where *B. xylophilus* was accidentally introduced at the beginning of the 20th century [9,10,11,12,13]. Japanese pine species were far more susceptible, and *B. xylophilus* expanded rapidly, resulting in extensive damages with reports of an annual loss rate in timber of 1.0 million m^3^ in the 70′s that peaked at 2.4 million m^3^ in 1979 [14,15]. Soon afterwards, *B. xylophilus* had spread to other neighboring Asian countries [16,17] that were confronted with similarly devastating impacts. In China, more than 1.7 million hectares had been affected by PWD until 2008, with more than 53 million trees dying out within a single year [18,19], whereas, in Korea, *B. xylophilus* is estimated to be causing annual losses of about 8 million US dollars [20] besides any additional costs for pest control and management [21].

In Europe, the pine wood nematode was first recorded in continental Portugal [22], and despite the containment measurements taken immediately [7], it subsequently expanded to Spain [23] and Madeira Island [24]. The estimated mortality risk of pine trees in southern Europe due to PWN is higher than 50%, something that could possibly have devastating effects on the European forests that occupy about 82 million hectares. The risk of *B. xylophilus* further spreading in Europe is even higher in areas where its insect vectors are present [25]. By 2030 the cumulative wood loss in the EU has been estimated at €22 billion representing 3.2% of the total value of PWN sensitive conifer trees [26].

Given the fact that high temperature and low humidity positively affect the spread and establishment of the PWD [10,27,28,29], coupled with the increase in international trade and movement of goods, the future impact of PWD is expected to increase [21,26], with Southern EPPO regions, in particular, exhibiting very high risk [30]. Situated at the eastern part of the Mediterranean basin, Greece can be readily included among the countries threatened most by a possible introduction of *B. xylophilus*, particularly as pine forests occupy a large proportion of Greek mainland and islands [31,32], its vector *M. galloprovincialis* Olivier 1795 is present [3] and Greece’s suitable climate conditions [33,34] favour the progress of the disease [35]. In this direction, numerous samples from all over the country are annually examined in the framework of the Greek national survey program against forest quarantine pests, in the attempt to promptly detect the pine wood nematode and inhibit its unimpeded expansion in forest ecosystems. Nevertheless, survey programs can simultaneously increase our basic knowledge on indigenous nematode species occurring in Greek forests as well, something very important given the limited number of studies on indigenous species [36]. Therefore, the purpose of this study is to document the indigenous nematode community that inhabits Greek conifer forests parallel to the Greek national survey program against quarantine pests, in this case, *B. xylophilus*.

## 2. Materials and Methods

In the framework of the National Survey Program regarding *B. xylophilus,* wood disc samples were collected from areas situated in Northern and Central Greece as well as the Northern Aegean islands. Samples were collected from fourteen regional units, namely eleven from Northern Greece (Halkidiki, Drama, Evros, Florina, Pella, Pieria, Rodopi, Serres, Thesprotia, Thessaloniki and Xanthi) two from Central Greece (Aitoloakarnania and Karditsa) and one from Northern Aegean islands (Lesvos). Samples were collected from phytosanitary inspectors during their regular inspections on permanent sites or emergency inspections at sites with weakened and dead trees. Wood disc samples were collected from the trunk at breast height and/or the branches, while at the same the location and the coordinates of each site were recorded. Finally, wood disc samples were sent and examined at the Forest Research Institute in Thessaloniki. In total, one hundred and twenty-three wood samples were collected from dying or diseased conifer trees. Samples were processed immediately after their arrival at the laboratory.

Nematodes were extracted using a modified Baermann funnel technique [37], and each sample contained about 10 gr of wood, cut into small to medium-sized pieces. Wood chips were wrapped in fine mesh and placed inside glass funnels of 100 mm in diameter. At the end of each funnel, a piece of soft silicone tube was attached to the stem. The tube was closed with a squeezer clip, and the funnel was then filled with water until it entirely covered the wood chips. Funnels were placed on a wooden custom-made stand appropriately designed for the extraction of multiple samples. Wood chips were soaked in water for at least 48 h at room temperature. The presence of nematodes was detected with the use of a binocular stereoscope (Zeiss SV8, 2× magnification zoom). Isolated nematodes were picked with a micropipette and mounted on a glass slide for further identification under a microscope (Zeiss Axio Imager A1, 10×–100× magnification).

Nematode identification was based on their morphological characteristics such as the stomodeum, reproductive organs, and tail morphology [8,38,39,40,41]. Nematodes were also assigned to trophic groups according to Yeates et al. [42], Scholze & Sudhaus [43], and Ferris [44]. Nematodes were identified at species level for the genus *Bursaphelenchus*, and at genus or family level for the rest of the nematodes recovered, while in some cases where deeper taxonomic identification was not possible, they were only classified according to their trophic group. Only nematode occurrence (presence/absence) in each sample was documented.

In order to detect any differences in pine nematode communities between regional units, Cluster analysis was performed based on the identified nematode taxa (species, genera, and families). Nematodes classified only into trophic groups were excluded from the analysis. Unweighted pair-group average (UPGMA) was used as a hierarchical clustering algorithm while distances were estimated using the Dice similarity index. Analysis was performed using PAST 3.0 [45].

Finally, sample distribution was depicted using QGIS Desktop 3.10.12 A Coruña, and the coordinates were projected using the Greek coordinate reference GGRS87. Greek coastline and regional units’ shapefiles were obtained from GEODATA.gov.gr (accessed on 10 February 2022) [46].

## 3. Results

**Figure 1:**
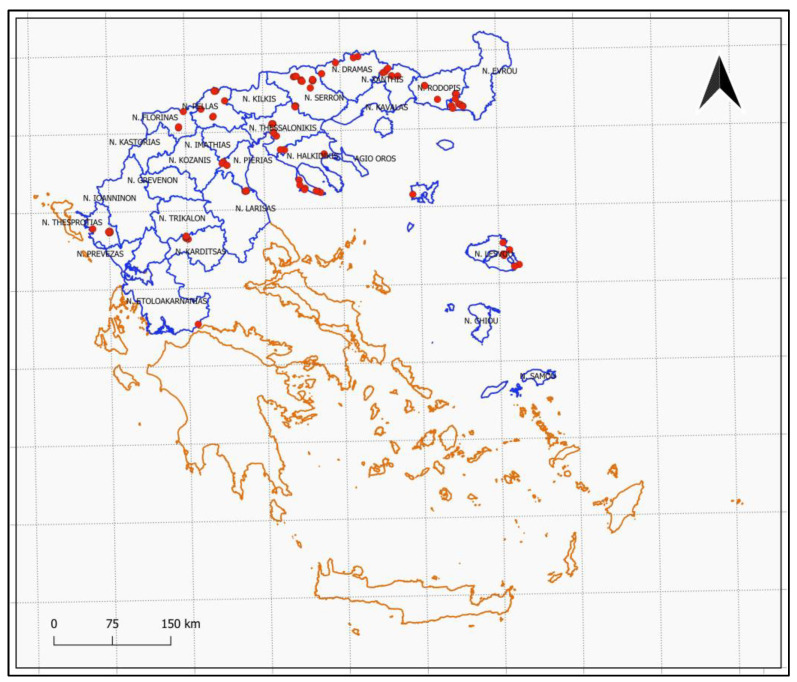
Sample distribution across the sampled areas.

Out of the 123 wood disc samples examined (Figure 1), nematodes were detected in 60 samples, i.e., 49% of the samples. *B. xylophilus* was not detected in any of the 60 samples, although other *Bursaphelenchus* spp. were detected in 35% of them (21 samples). Among those samples, 17 samples contained only one *Bursaphelenchus* species (81% of the samples), while the rest of the samples contained two *Bursaphelenchus* species. At the same time, the majority of wood disc samples with nematodes (95% of the samples) contained other nematode taxa together with *Bursaphelenchus* species.

In particular, four *Bursaphelenchus* species were identified: *Bursaphelenchus hellenicus* Skarmoutsos, Braasch, Michalopoulou 1998, *B. leoni* Baujard 1980, *B. mucronatus* Mamiya and Enda 1979 and *B. sexdentati* Rühm 1960. In addition to them, there were also some *Bursaphelenchus* spp. individuals that could not be assigned to a specific species (Table 1) due to either their premature stage and/or the condition of their body. *B. hellenicus* was the most abundant species followed by *B. mucronatus* and *B. sexdentati* with equal frequency, *B. leoni* was the least encountered species.

Regarding the other nematode taxa detected in the wood disc samples (Table 1), they can also be categorized into trophic groups as follows: *Aphelenchoides* sp., *Aphelenchus* sp., *Tylencholaimellus* sp. (fungivores), *Diplenteron* sp., *Eucephalobus* sp., *Panagrobelus* sp., *Panagrolaimus* sp., *Plectus* sp., *Pristionchus* sp., *Rhabditis* sp., *Rhodolaimus* sp. (bacterivores), *Devibursaphelenchus* sp., *Ektaphelenchus* sp., *Clarkus* sp. (predators), *Thonus* sp. (predator/omnivore) and *Parasitorhabditis* sp. (entomophilic). *Laimaphelenchus* sp. is classified in more than one feeding group as it includes non-parasitic plant feeding, fungivorous as well as predatory nematodes. Similarly, although *Devibursaphelenchus* sp. is classified as fungivorous by Ferris [44], it has also been reported predating on other nematodes [47,48]. Some individuals were identified to family level e.g., Anguinidae (fungivores/plant feeders), Tylenchidae (non-parasitic plant feeders), Dolichodoridae (plant parasitic), and Rhabditidae (bacterivores), while some others were separated only after their feeding group based on the structures of the mouthparts.

Nematodes were detected in the wood of the following conifer species: *Abies borisii-regis* Mattfeld, *P. brutia* Tenore, *P. halepensis* Miller, *P. maritima* Aito, *P. nigra* Arnold, and *P. sylvestris* Linnaeus, as well as unspecified *Pinus* species (Table 1).

**Figure 2:**
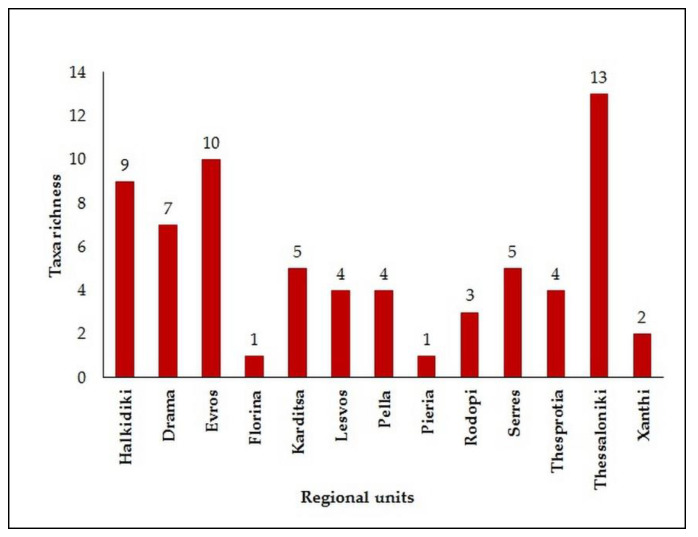
Nematode taxa richness per regional unit.

In total, nematodes were detected in overall thirteen out of fourteen regional units, namely Halkidiki, Drama, Evros, Florina, Karditsa, Lesvos, Pella, Pieria, Rodopi, Serres, Thesprotia, Thessaloniki, and Xanthi (Figure 1 and Figure 2). Among them, Thessaloniki and Evros were the two regions with the highest number of wood disc samples with nematode presence, and at the same time, these areas exhibited also the highest number of nematode taxa, followed by Halkidiki and Drama (Figure 2). Except for Florina, Pieria, and Serres where no *Bursaphelenchus* spp. were detected at all, wood disc samples from every other area contained both *Bursaphelenchus* species and other nematode taxa (Table 1).

**Figure 3:**
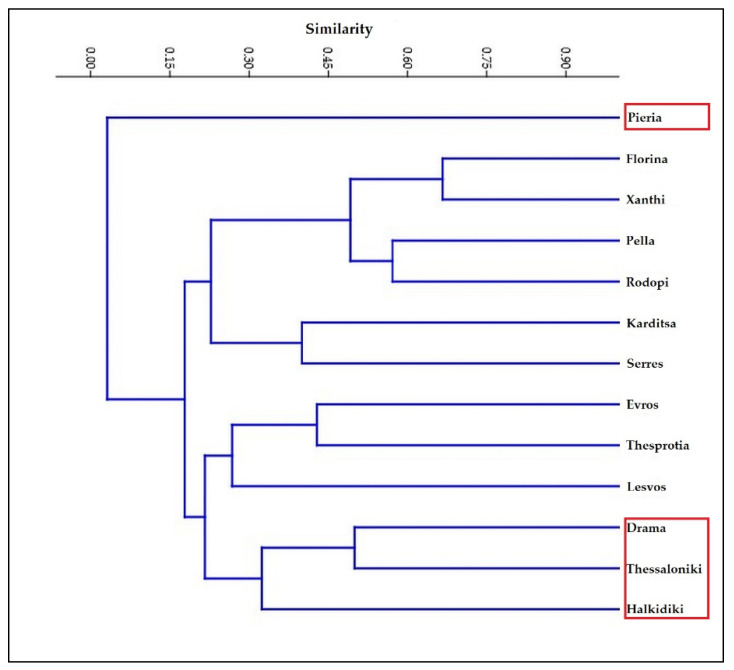
Cluster analysis on nematode taxa.

Cluster analysis based on the occurrence of nematode taxa (Figure 3) resulted in relatively heterogenous clusters with the exception of the marked cluster that includes Drama, Thessaloniki, and Halkidiki. Moreover, Pieria is distinctly separated from all other regions.

## 4. Discussion

In the current study, nematodes and their communities in pine forests were systematically examined and recorded for the first time in Greece, enhancing significantly our basic knowledge of the indigenous nematode fauna. The study was conducted alongside the annual survey programme against harmful organisms, in this case, *B. xylophilus*. *B. xylophilus* was not detected in any of the wood samples examined. In general, the introduction of PWN in Greece through natural dispersal is not very likely since *Monochamus* spp. beetles, PWN insect vector, cover relatively short distances [3,49]. This fact, however, does not significantly reduce the risk of PWN invading Greece, as international trade and transport of wood products is considered to be the main pathway of *B. xylophilus* invasion and expansion [50,51], especially when *B. xylophilus* and its vector are introduced together [51]. In spite of the attempts to ensure proper treatment or monitoring of wood products, materials infested with *B. xylophilus* and/or its insect vector are being regularly recorded worldwide at points of entry, such as ports [52], even from countries known to be PWN-free [4,53]. For example, in Portugal, *B. xylophilus* presence is consistently recorded in areas around ports that are associated with the trade of goods [4]. Greece’s ports as possible entry points for *B. xylophilus* are among the ones’ that require high priority surveillance in order to prevent a rapid invasion of *B. xylophilus* and pine wilt disease across Europe [54].

Greece, like many other EPPO countries, is considered a risk area for the introduction and establishment of *B. xylophilus*, given the abundance of its host trees coupled with the occurrence of its insect vector [6]. Out of the seven indigenous *Pinus* spp. in Greece [31] four are susceptible to PWD: *P. halepensis, P. nigra, P. pinea* Linnaeus, *P. sylvestris*. In fact, *B. xylophilus* can be found in almost any conifer species (except *Thuja* and *Taxus* spp.) weakened enough to allow *Monochamus* species to oviposit and transmit the nematode in addition to pine species that express pine wilt disease [55].

Additionally, climatic conditions in Greece further favour a possible establishment of *B. xylophilus*. Average summer temperatures in the Mediterranean regions are high enough to support pine wilt disease in susceptible trees [56]. In Greece, the lowest minimum summer temperature is 20 °C [57], ideal for the development of both *B. xylophilus* and *Monochamus* spp. and consequently the expression of pine wilt disease. Both the nematode and its insect vector strongly depend on temperature. In fungal cultures of *Botrytis cinerea* Persoon (1794), the postembryonic development of *B. xylophilus* requires 12, 6, 4–5, and 3 days at 0 °C, 15 °C, 21 °C, 26 °C, and 30 °C, respectively [58], while it reproduces in 12 days at 15 °C, 6 days at 20 °C and 3 days at 30 °C [56]. *M. galloprovincialis* larval development is also dependent on temperature. There is a linear relationship between temperature and development duration in days between 15 °C and 30 °C [59]. However, the developmental rate seems to decrease above 30 °C for both PWN and pine sawyer beetles [56,59], although areas with climatic conditions that do not favour the expression of the disease could possibly act as reservoirs. [6,50].

The natural dispersal of *B. xylophilus* between host trees occurs primarily during the maturation feeding of *Monochamus* (Coleoptera, Cerambycidae) species. Even though the main vector of PWN in Europe is *M. galloprovincialis* [60], and this species occurs widely yet in low population levels in Greece [61], there is always the risk of accidentally introducing non-native sawyer beetles [62,63]. Besides *M. galloprovinciallis* in Europe and *M. carolinensis* Olivier 1792 in North America or *M. alternatus* Hope 1842 in East Asia, many other *Monochamus* species have been reported capable of carrying *B. xylophilus* [3,49,64]. The remarkable biological similarities among *Monochamus* species globally, render many of these species putative vectors of *B. xylophilus*, particularly in the presence of their host trees [49]. Even though it is still not clear whether *Monochamus* species can directly cause tree mortality, infestation by the pine sawyer beetle is definitely weakening tree physiology, making it more susceptible to other secondary pests and diseases that ultimately lead to significant timber degradation and economic losses [3,51,65,66].

Moreover, besides *Monochamus* species as vectors of *B. xylophilus*, PWN has been found in association with other Coleoptera species such as *Acanthocinus griseus* Fabricius 1793, *A. gundaiensis* Kano 1933, *Amniscus sexguttatus* Dillon 1956, *Arhopalus rusticus* Linnaeus 1758, *Aromia bungii* Faldermann 1835, *Asemum striatum* Linnaeus 1758, *Corymbia succedanea* Hua 2002, *Neacanthocinus obsoletus* Olivier 1795, *N. pusilus* Kirby 1837, *Spondylis buprestoides* Linnaeus, 1758, *Uraecha bimaculata* Thomson 1864, *Xylotrechus sagittatus* Germar 1821, *Hylobius pales* Herbst 1797, *Odontotermes formosanus* Shiraki 1909, *Pissodes approximates* Hopkins 1911, *Tomicus piniperda* Linnaeus 1758, as well as other genera (e.g., *Acalolepta* sp., *Chrysobothris* sp., *Rhagium* sp.). However, there is still no evidence that any of these species can act as vectors of the nematode in nature [3,56,67].

Even though *B. xylophilus* was not identified among the nematode species retrieved from the wood disc samples, four *Bursaphelenchus* spp., *B. hellenicus*, *B. leoni*, *B. mucronatus,* and *B. sexdentati*, were detected in about half of the samples with nematode presence indicating a strong occurrence of this genus in pines. *B. hellenicus*, *B. mucronatus*, *B. leoni,* and *B. sexdentati,* as well as *B. eggersi* Rühm 1956 and *B. vallesianus* Braasch, Schönfeld, Polomski, Burgermeister 2004 have already been documented in Greece [32,68,69]. However, *B. eggersi* [32] a member of the *eggersi* group [38,40,41,70] and *B. vallesianus* [69], a member of the *sexdentati* group [38,40,41,70], were not detected in the present study. In general, *B. mucronatus* and *B. sexdentati* are acknowledged as the most abundant species in Europe, with the latter being more frequent in the southern European regions [70,71]. In contrast, *B. leoni* is recognized as a typical Mediterranean species, based on their dispersal and frequency, although they have also been occasionally found in Central Europe [68]. Finally, *B. hellenicus* exhibits the most limited natural range, which contains only two other countries, namely Italy [72] and Turkey [73] besides Greece [32,74]. Additionally, in terms of pathogenicity, *B. mucronatus*, *B. vallesianus,* and *B. sexdentati* have been characterized to be highly pathogenic [36,75,76] although such findings have not been confirmed under natural forest stand conditions [77], and the expression of virulence could also be dependent on host susceptibility as shown by Carropo et al. [78]. *B. leoni* was found to be less pathogenic whereas, *B. helleniccus* is considered to be non-pathogenic [36,68].

Most of the aforementioned *Bursaphelenchus* species have also been documented in Greece’s neighboring and surrounding countries (Table 2). For example, in addition to *B. leoni* and *B. sexdentati, B. idius* Rühm 1956 have also been recovered from weakened trees in Cyprus [79,80]. Similarly, *B. anamurius* Akbulut, Braasch, Baysal, Brandstetter, Burgermeister 2007, *B. pinophilus* Brzeski, and Baujard 1997 and *B. vallesianus* are already known to occur in Turkey, besides *B. hellenicus*, *B. mucronatus,* and *B. sexdentati* [73,81,82,83,84]. On the other hand, species richness of *Bursaphelenchus* spp. in Italy is considerably higher, with numerous other *Bursaphelenchus* species (e.g., *B. abietinus* Braasch and Schmutzenhofer 2000, *B. andrassyi* Dayi, Calin, Akbulut, Gu, Schröder, Vieira, Braasch 2014, *B. eremus* Rühm 1956, *B. fraudulentus* Rühm 1956, *B. fungivorous* Franklin and Hooper 1962, *B. minutus* Walia, Negi, Bajaj, Kalia 2003 and *B. tusciae* Ambrogioni and Palmisano 1998) having been identified [72,85,86,87,88] besides the ones already known in Greece [72,89], something that needs particular attention given the strong commercial relationships.

As more than one *Bursaphelenchus* species were found in almost 20% of the wood disc samples inhabited by nematodes, it can be easily deduced that a single tree can host more than one species at the same time. This is something that has also been reported in the past, with up to four different *Bursaphelenchus* species co-existing in one tree [70,89]. Furthermore, Penas et al. [90] have verified that one insect vector could possibly carry several *Bursaphelenchus* species, while one *Bursaphelenchus* species can have different insect vectors [8,70], suggesting a non-specific relationship between insect vectors and *Bursaphelenchus* spp. [90]. As a consequence, both these mechanisms could explain and maintain the co-existence of different *Bursaphelenchus* spp. in a single tree. Several insect species can carry *Bursaphelenchus* nematodes acting as vectors, mainly longhorn beetles (Cerambycidae), bark beetles (Curculionidae-Scolytinae), and jewel beetles (Buprestidae) [8,70,91,92]. For example, *B. mucronatus* was found to be associated with *Ips sexdentatus* Börner 1776, while *B. sexdentati* was associated with *Orthotomicus erosus* Wollaston 1857, *Acanthocinus aedilis* Linnaeus 1758, and *Arhopalus rusticus* Linnaeus 1758 [93]. On the other hand, insect species are capable of vectoring more than one *Bursaphelenchus* species, e.g., *O. erosus* carried three different *Bursaphelenchus* spp., *Hylurgus ligniperda* Fabricius 1787 two *Bursaphelenchus* species and both *Tomicus piniperda* and *I. sexdentatus* one *Bursaphelenchus* species each. [94].

To elucidate further the behavior and occurrence of *Bursaphelenchus* spp. within a host tree, sampling effort should aim at screening different parts of the same tree. Even though *Bursaphelenchus* species have been recovered from all parts of the tree, occurrence frequencies can differ [95]. Specifically, *Bursaphelenchus* spp. have been detected both in the stem and the branches, with numbers greater in the lower part of the stem compared to branches, whereas they have been detected even in the roots [75,96]. Similar findings have also been reported for *B. xylophilus* on several occasions. For instance, it has been shown that *B. xylophilus* nematodes migrate within infected trees soon after the initial infection or inoculation [97,98]. Trunk samples had significantly higher nematode density levels than the branches, as *B. xylophilus* nematodes moved from the infected branches to the stem after infestation [99,100,101].

Apart from *Bursaphelenchus* spp., other nematode taxa were also recovered in many wood disc samples. The retrieved taxa belong to different trophic groups, ranging from bacterivores and fungivores, to phytophagous and predatory nematodes, most of which have never been recovered from wood disc samples in Greece before. Many of the genera recorded, apart from *Bursaphelenchus* spp., belong to families that are typical of environments with nutrient availability (Rhabditidae, Panagrolaimidae) or to families adapted to stress with a wide ecological range (Cephalobidae, Aphelenchidae, Aphelenchoididae, Anguinidae). On the other hand, Mononchidae and Quadsianematidae are more sensitive to disturbance and are commonly present in more stable environments [102]. Nevertheless, the presence of many different groups of nematodes appears feasible given the great variety of available resources as it is suggested by Moll et al. [103].

Many of the free-living nematode taxa recovered in the present study have also been reported to be associated with insects, in addition to their initial trophic group assignment [42,104,105,106] such as members of the families Aphelenchoididae, Rhabditidae, Neodiplogasteridae, and Panagrolaimidae [42,95,107]. *O. erosus, H. ligniperda*, *T. piniperda,* and *I. sexdentatus*, as well as *Hylastes linearis* Erichson 1836 and *Pissodes castaneus* De Geer 1775, have all been found to carry nematodes belonging to different genera of the Aphelenchoididae family or other taxonomic groups. As already mentioned, *O. erosus, H. ligniperda*, *T. piniperda,* and *I. sexdentatus* also carried members of the genus *Bursaphelenchus* [94]. Therefore, it is not uncommon for many nematode species to co-exist in a single host, as has been demonstrated in previous studies [75,80,83,89,108]. For example, Caroppo et al. [89] recorded the co-occurrence of Rhabditida, Aphelenchida, and Tylenchida nematodes, while Đođ et al. [109] found that saprophytic nematodes such as Rhabditidae, Diplogasteridae, and Cephalobidae were found to be dominant but also co-existing with low density populations of *Bursaphelenchus* spp. Similarly, numerous other nematode genera were found together with the *Bursaphelenchus* species recovered from *Pinus pinaster* Aiton trees in Portugal [100].

In order to investigate whether nematode communities from different areas differ from each other, a Cluster analysis was performed. The analysis was based on all reported taxa i.e., both on *Bursaphelenchus* spp. and the other documented nematode taxa (genera and families), and resulted in the formation of rather heterogenous groups. One would expect that areas with close proximity to each other would group together as in the case of Drama, Thessaloniki, and Halkidiki (Figure 3), which could mean that there is a great possibility that these areas share similar nematode communities, although this could not be verified at the present time. At present, it seems that the different areas examined are classified based on nematode taxa richness rather than community composition.

Many factors affect both nematode presence and community structure such as tree species, environmental variables, as well as the time of the year that sampling took place. For example, Moll et al. [103] who studied nematode communities from deadwood of 13 different tree species came to the conclusion that nematode composition was strongly related to tree species as well as the presence of other co-occurring biota such as fungi and prokaryotes. As already mentioned, environmental variables play an important role in nematode community composition. For instance, soil nematode communities appear to differ across different latitudes [110] while climate variables such as temperature and precipitation are strongly related to nematode community structure and composition [111,112]. Finally, even the time of sampling during the year could also be an important factor influencing nematode community studies since nematode community composition tends to differ among seasons [113].

Environmental traits such as temperature and humidity, as already mentioned, are factors of essential importance influencing the manifestation of PWD. Nematode infection of a healthy pine tree occurs from early June to late July, coinciding with the period of maturation feeding of adult pine sawyers when high temperature and low humidity promote the progress of PWD [10]. As Ichihara et al. [28] have shown, temperature affects migration patterns of *B. xylophilus* in the tissues of *Pinus thunbergii* Parlatore and the expression of PWD. Estimated optimal temperatures that PWD progresses have been reported to range from 25 °C to 30 °C [27,29].

Relative to climate, climate change is a great concern regarding *B. xylophilus* expansion into other countries and continents. As climate changes the distribution of PWN is expected to expand along with the expression of the disease. Different global scenarios predict the expansion of PWN risk areas globally even in areas that are currently not suitable for the expression of the disease [114]. For instance, future climatic scenarios predict that by 2030 there will be a significant increase in the distribution of PWD across Europe ranging from 8% up to 34% of its total area, or even up to 55% under even more extreme scenarios [54]. As a result, the predicted changes in habitat suitability for the potential host trees (e.g., *Pinus sylvestris*) would additionally impair the physiology of these trees, rendering them more susceptible to pests and pathogens. This could ultimately alter the current PWD risk areas into high-risk areas in the near future [114]. In general, it can be easily deduced that as climate change progresses, both the intensity and the expansion of PWD is expected to increase, leading to even greater economic damages [21].

## 5. Conclusions

In summary, it can be easily deduced that in the framework of the national survey programs focusing on *B. xylophilus*, significant knowledge can be gained and accumulated regarding other *Bursaphelenchus* and nematode species as well. One-year observations and screening of samples from the northern and central parts of Greece have already resulted in the record of four different indigenous *Bursaphelenchus* species, coupled with the identification of 24 additional nematode taxa, enhancing significantly our knowledge of the poorly studied nematode species inhabiting pine forests in Greece.

Further future investigation, covering greater parts of Greece, even the whole Greek domain, together with the employment of molecular techniques will provide significant and more complete and accurate information regarding indigenous *Bursaphelenchus* spp. as well as the rest of the local coniferous nematode fauna.

## Figures and Tables

**Table 1:** Tree species, *Bursaphelechus* spp. and other nematode taxa detected per regional unit and locality.

Regional Unit	Locality	Tree Species	*Bursaphelenchus* spp.	Other Nematode spp.
Halkidiki	Kassandra	*Pinus halepensis*	*B. leoni*	*Aphelenchus* sp.
		*Pinus halepensis*	*B. mucronatus*	*Devibursaphelenchus* sp.
		*Pinus halepensis*		*Diplenteron* sp.
		*Pinus halepensis*		*Parasitorhabditis* sp.
		*Pinus halepensis*		*Panagrolaimus* sp.
		*Pinus halepensis*		*Pristionchus* sp.
		*Pinus halepensis*		*Thonus* sp.
Drama	Drama	*Pinus* sp.		*Parasitorhabditis* sp.
		*Pinus* sp.		Tylenchidae
	Neurokopi	*Pinus* sp.	*B. mucronatus*	Bacterivore
		*Pinus sylvestris*	*B. hellenicus*	*Aphelenchoides* sp.
		*Pinus sylvestris*	*B. leoni*	Anguinidae
Evros	Alexandroupoli	*Pinus brutia*		*Ektaphelenchus* sp.
		*Pinus brutia*		*Panagrolaimus* sp.
		*Pinus brutia*		*Parasitorhabditis* sp.
		*Pinus brutia*		*Rhabditis* sp.
		*Pinus brutia*		Anguinidae
		*Pinus* sp.	*B. hellenicus*	*Clarkus* sp.
		*Pinus* sp.	*Bursaphelenchus* sp.	*Eucehpalobus* sp.
		*Pinus* sp.		*Laimaphelenchus* sp.
		*Pinus* sp.		*Parasitorhabditis* sp.
		*Pinus* sp.		Plant parasitic
Florina	Florina	*Pinus nigra*		*Laimaphelenchus* sp.
Karditsa	Mouzaki	*Pinus brutia*	*Bursaphelenchus* sp.	*Aphelenchoides* sp.
		*Pinus brutia*		*Rhabditis sp.*
		*Pinus brutia*		Anguinidae
		*Pinus brutia*		Dolicodoridae
Lesvos	Lesvos	*Pinus brutia*	*B. hellenicus*	*Eucehpalobus* sp.
		*Pinus brutia*	*B. sexdentati*	*Plectus* sp.
Pella	Aridaia	*Abies borisii-regis*	*B. mucronatus*	
		*Pinus* sp.	*Bursaphelenchus* sp.	*Aphelenchoides* sp.
		*Pinus* sp.		*Laimaphelenchus* sp.
	Pella	*Pinus sylvestris*		*Laimaphelenchus* sp.
Pieria	Pieria	*Pinus nigra*		*Panagrolaimus* sp.
Rodopi	Rodopi	*Pinus maritima*	*B. hellenicus*	*Laimaphelenchus* sp.
		*Pinus maritima*	*B. mucronatus*	Bacterivore
Serres	Sidirokastro	*Pinus brutia*		*Laimaphelenchus* sp.
		*Pinus brutia*		*Merlinius* sp.
		*Pinus brutia*		Anguinidae
		*Pinus brutia*		Dolichodoridae
		*Pinus brutia*		Rhabditidae
Thesprotia	Thesprotia	*Pinus* sp.	*B. hellenicus*	*Clarkus* sp.
		*Pinus* sp.	*Bursaphelenchus* sp.	*Tylencholaimellus* sp.
Thessaloniki	Lagkadas	*Pinus* sp.	*Bursaphelenchus* sp.	*Laimaphelenchus* sp.
		*Pinus* sp.		*Parasitorhabditis* sp.
		*Pinus* sp.		*Rhodolaimus* sp.
	Thessaloniki	*Pinus maritima*	*B. sexdentati*	*Aphelenchus* sp.
		*Pinus maritima*		*Laimaphelenchus* sp.
		*Pinus maritima*		*Panagrobelus* sp.
		*Pinus maritima*		*Plectus* sp.
		*Pinus* sp.	*B. hellenicus*	*Aphelenchoides* sp.
		*Pinus* sp.	*B. leoni*	*Merlinius* sp.
		*Pinus* sp.	*B. sexdentati*	*Parasitorhabditis* sp.
		*Pinus* sp.	*Bursaphelenchus* sp.	Anguinidae
Xanthi	Xanthi	*Pinus sylvestris*	*Bursaphelenchus* sp.	*Laimaphelenchus* sp.
		*Pinus sylvestris*		Bacterivore

**Table 2:** *Bursaphelenchus* spp. records in Greece and neighboring countries (●) indicates presence ^1^.

*Bursaphelenhus* spp.	Cyprus	Greece	Italy	Turkey
*B. abietinus*			●	
*B. anamurius*				●
*B. andrassyi*			●	
*B. eremus*			●	
*B. fraudulentus*			●	
*B. fungivorous*			●	
*B. hellenicus*		●	●	●
*B. idius*	●			
*B. leoni*	●	●	●	
*B. minutus*			●	
*B. mucronatus*		●	●	
*B. pinophilus*				●
*B. sexdentati*	●	●	●	●
*B. tusciae*			●	
*B. vallesianus*		●		●

^1^ For references see text.

## Data Availability

The data presented in this study are available on request from the corresponding author.
